# The power lies in the glycans

**DOI:** 10.7554/eLife.102427

**Published:** 2024-09-20

**Authors:** Luca Unione, Jesús Jiménez-Barbero

**Affiliations:** 1 Center for Cooperative Research in Biosciences, Basque Research and Technology Alliance Derio Spain; 2 Ikerbasque, Basque Foundation for Science Bilbao Spain; 3 Department of Organic & Inorganic Chemistry, Faculty of Science and Technology, University of the Basque Country Leioa Spain; 4 Centro de Investigacion Biomedica En Red de Enfermedades Respiratorias Madrid Spain

**Keywords:** glycans, natural killer cells, immunotherapy, antibodies, Other

## Abstract

Glycans play an important role in modulating the interactions between natural killer cells and antibodies to fight pathogens and harmful cells.

**Related research article** Kremer P, Lampros E, Blocker A, Barb A. 2024. One N-glycan regulates natural killer cell antibody-dependent cell-mediated cytotoxicity and modulates Fcγ receptor IIIa/CD16a structure. *eLife* **13**:RP100083. doi: 10.7554/eLife.100083.

Since the COVID-19 pandemic, antibodies have become a household name. These Y-shaped proteins are part of the immune system and recognize, tag and neutralize pathogens, as well as cancer cells. Each tip of the Y of the antibody (the 'arms') can bind to target cells, while the vertical 'leg' of the Y communicates with other components of the immune system.

Antibodies can help neutralize harmful cells through a mechanism known as antibody-dependent cell-mediated cytotoxicity (ADCC). During this process, antibodies coating a target cell bind to Fcγ receptors on the surface of an immune cell called a natural killer cell. This event activates the natural killer cell, causing it to release molecules that kill the target cell.

For over four decades, antibodies have been successfully cloned and engineered in laboratories to make monoclonal antibodies as targeted treatments for various diseases, including cancer (Sharma et al., 2023). Strengthening the interactions between Fcγ receptors and antibodies has been shown to improve the immune response through ADCC and thus represents a strategy to boost therapeutic efficacy (Presta et al., 2002).

Both Fcγ receptors and antibodies are glycoproteins. These are proteins that have undergone a process called glycosylation that results in the attachment of carbohydrate chains (known as glycans). Fcγ receptors have five glycosylation sites on their extracellular portion, two of which are close to where antibodies bind (Reily et al., 2019). The glycan composition at each of these sites is usually diverse. Previous research found that the specific glycan composition of natural killer cells (and possibly of their receptors) affects the potency of ADCC (Rodriguez Benavente, 2024; Rodriguez Benavente et al., 2023). However, so far, it has been unclear how this enhances ADCC. Now, in eLife, Paul Kremer, Elizabeth Lampros, Allison Blocker and Adam Barb at the University of Georgia report how the glycan composition at a specific glycosylation site on the Fcγ receptor modulates ADCC (Kremer et al., 2024).

Kremer et al. generated a library of Fcγ receptors with various mutations to the area that binds antibodies and with different glycoforms (Figure 1). This allowed them to assess how individual glycans and their location within the Fcγ receptor affect the ability of natural killer cells to bind to antibodies. This exhaustive approach revealed that the glycan chain attached to the Fcγ receptor at a specific amino acid site (known as N162) is a critical mediator of the increased ADCC responses, and that shorter glycans at this site significantly increase the affinity for the antibody (Figure 1).

**Figure 1.**
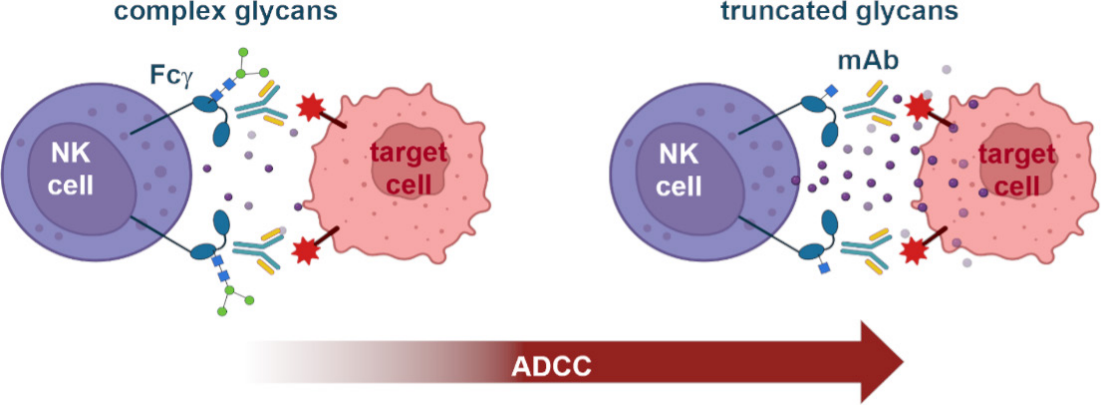
Schematic illustrating the binding affinity of Fcγ receptors and antibodies. Natural killer cells (NK cell, purple) express Fcγ receptors on their surface (teal structures) that contain glycan units (structure made out of dots and squares). The Fcγ receptors bind to antibodies (blue and yellow Y-shaped structure) that coat target cells (pink), triggering natural killer cells to release molecules (small purple circles) that kill the target cell. A strong connection between Fcγ receptors and antibodies enhances this process, known as antibody-dependent cell-mediated cytotoxicity (ADCC). Kremer et al. show that the complexity of glycans in the Fcγ receptor affects ADCC potency. Fcγ receptors with more complex glycans (left) induce weaker ADCC than those with removed glycan units (truncated, right). In particular, the affinity between the receptor and the antibody increases with each glycan truncation step. mAb: monoclonal antibody. Figure 1 was created with BioRender.com.

Next, the team evaluated how the composition of the identified glycan affects the structure and flexibility of Fcγ receptors. New experimental methodologies based on atomic resolution techniques, such as nuclear magnetic resonance, provided key structural insights into understanding the mechanisms underlying the antibody-Fcγ binding event. With the support of computer studies, Kremer et al. concluded that a unique composition of the glycan at this site enhances antibody binding by stabilizing the conformation of Fcγ required for a stronger interaction with the antibody.

The study of Kremer et al. provides the first evidence that specific glycosylation on the Fcγ receptor is responsible for a higher binding affinity between the receptor and the antibody, building on existing evidence of how antibody glycosylation affects Fcγ-binding affinity (Shields et al., 2002; Ferrara et al., 2011; Mizushima et al., 2011). It demonstrates that Fcγ engineering is a promising strategy to promote improved ADCC responses through efforts to tune the glycan composition of natural killer cells. In that sense, natural killer cell-based immunotherapies have already shown promising outcomes in cancer treatment (Page et al., 2024). The results presented by Kremer et al. indicate an unexplored avenue to improve natural killer cell-mediated immunotherapies in a variety of diseases that may eventually improve patient responses.
